# Effect of mild-intensity exercise training with capsiate intake on fat deposition and substrate utilization during exercise in diet-induced obese mice

**DOI:** 10.20463/pan.2020.0014

**Published:** 2020-09-30

**Authors:** Deunsol Hwang, Jong-beom Seo, Jisu Kim, Kiwon Lim

**Affiliations:** 1 Department of Physical Education, Konkuk University, Seoul, Republic of Korea; 2 Department of Sports Medicine and Science, Konkuk University, Seoul, Republic of Korea; 3 Physical Activity and Performance Institute (PAPI), Konkuk University. Seoul, Republic of Korea

**Keywords:** anti-obesity, fat oxidation, CHO oxidation, RER, mild-intensity exercise, high-fat, capsiate, capsaicin

## Abstract

**[Purpose]:**

While the anti-obesity effects of exercise and capsiate are well-observed individually, the effect of exercise with capsiate intake has not been systematically explored yet. Therefore, the purpose of this study is to investigate whether the anti-obesity effects of exercise training can be further enhanced by capsiate intake.

**[Methods]:**

8-week-old male mice were divided into 3 groups (*n* = 8 per group): sedentary group (SED; nontrained), exercise-trained group (EXE) and exercisetrained group with 10 mg/kg of capsiate intake (EXE+CAP). All mice were offered high-fat diet and water ad libitum. The mild-intensity treadmill training was conducted 5 times a week for 8 weeks. After 8 weeks, metabolism during exercise and abdominal fat weight were measured.

**[Results]:**

Body weight and the rate of total abdominal fat were significantly less in EXE+CAP than in SED but not between EXE and SED. The average of respiratory exchange rate during exercise was significantly much lower in EXE+SED (*p* = 0.003) compared to the difference between EXE and SED (*p* = 0.025). Likewise, the fat oxidation during exercise was significantly much higher in EXE+SED (*p* = 0.016) compared to the difference between EXE and SED (*p* = 0.045). Then, the carbohydrate oxidation during exercise was significantly much lower in EXE+SED (*p* = 0.003) compared to the difference between EXE and SED (*p* = 0.028).

**[Conclusion]:**

In conclusion, the anti-obesity functions of exercise training can be further enhanced by capsiate intake by increasing fat oxidation during exercise. Therefore, we suggest that capsiate could be a candidate supplement which can additively ameliorate obesity when combined with exercise.

## INTRODUCTION

Obesity causes endocrine imbalances which can lead to various cardiovascular diseases and type 2 diabetes [1]. This is because excess adipose tissue actively releases a large amount of cytokines and bioactive mediators such as leptin, adiponectin, and interleukin-6 [2]. Obesity increases the risk of mortality [3,4] and is a persistent global health problem [5]. Thus, considerable efforts to treat obesity and obesity-related problems have been exerted globally.

**Fig. 1.**
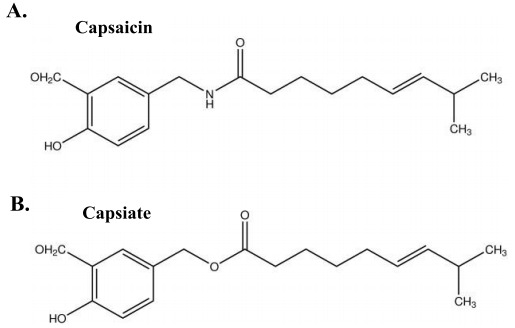
The chemical structure of capsaicin (A) and capsiate (B).

Capsiate, which was discovered in ‘CH-19 sweet pepper,’ is a capsaicin analogue [6] (Figure 1). While capsaicin has a strong, spicy taste and tends to cause stomachaches [7,8], capsiate is not pungent [9] but can increase energy expenditure to an equal extent as capsaicin [10,11]. In previous studies, acute administration of capsiate significantly increased oxygen uptake at rest [11,12], blood norepinephrine concentrations [12], and activity of the sympathetic nervous system [13]. Moreover, capsiate intake for 2 weeks significantly reduced body weight, body fat percentage [14], and abdominal fat [11,15] in humans and rodents. Thus, capsiate has been considered a possible supplement that can ameliorate obesity [16,17].

Exercise is one of the primary and most efficient methods to manage obesity [18]. Exercise is not only a calory-burning activity itself but it also increases fat-free mass [19] and energy expenditure level at rest [20], which leads to an energy imbalance toward weight loss. Moreover, a very important feature of exercise training is that it can enhance the fat oxidation capacity during exercise [21,22] so that fat is preferentially oxidized over carbohydrates as a fuel source even when an equal volume of exercise is performed at an equal absolute exercise intensity [23]. Therefore, many studies are being conducted on constructing a form of exercise training that can consume more fat [24,25].

While it is well-observed that both exercise and capsiate have antiobesity effects, the effect of exercise with capsiate intake has not been elucidated. There was only one study that investigated the combinatorial effects of exercise and capsiate, but there were some limitations as this previous study did not strictly control the total amount of capsiate intake and exercise training [26]. On the contrary, the weight-loss effects of exercise with capsaicin were well documented [27–29]. Additionally, many nutritional supplements such as caffeine and green tea extract have been shown to enhance fat oxidation and improve endurance performance [30], however, there has been no study that has investigated the effects of exercise training with capsiate on substrate utilization during exercise. Therefore, the purpose of this study is to investigate whether the anti-obesity effects of exercise training can be further enhanced by capsiate intake according to a change of substrate utilization during exercise in diet-induced obese mice.

## METHODS

### Animal care

This study used male 8-week-old ICR mice. Before the study began, they were adapted to the laboratory environments. All mice were housed in standard plastic cages (4 mice per cage) under controlled humidity (50 ~ 55 %), temperature (23 ℃ ± 1 ℃) and lighting (12:12-hr lightdark cycle; lights on at 08:00 am) conditions with highfat (60 % fat of total kcal) diet (HFD; Research Diets, Inc., New Brunswick, NJ, USA) and water available *ad libitum*. This study was approved by Konkuk University Institutional Animal Care and Use Committee.

### Study design

Mice were randomly divided into three groups (*n* = 8 per group): sedentary group (SED; non-trained), exercise-trained group (EXE) and exercise-trained group with 10 mg/kg of capsiate intake (EXE+CAP). SED and EXE were also orally administered the equal solvent without capsiate for 8 weeks.

Exercise training intensity was set at mild condition (about 60 % VO_2max_) to prevent concealing of capsiate effect from the training. The training using treadmill was conducted by 5 times a week for 8 weeks. To avoid decreasing relative exercise intensity, absolute exercise intensity had been gradually increased (Table 1). The mice were orally administered capsiate 30 min before training.

### Body weight, food intake and abdominal adipose tissue measurement.

Body weight (BW) and food intake were measured every day. At the end of the experiment, epididymal, perirenal and mesenteric fat were surgically obtained from the deeply anesthetized mice. Total abdominal fat was calculated from the sum of epididymal, perirenal and mesenteric fats. All tissues were weighed just after being dissected and subsequently stored at -80 ℃.

### Metabolic analysis during exercise[31]

To investigate the effect of exercise training with capsiate intake on substrate utilization during exercise, metabolic analysis during exercise was conducted on the last weekend of the experiment for an hour at 13 m/min while fixed at 8 ° slope. The mice were orally administered capsiate 30 min before measurement. Mice were solely separated one by one in the metabolic treadmill chamber to measure energy metabolism during exercise. Respiratory gas (O_2_ uptake and CO_2_ production) was analyzed by a mass spectrometer (model RL-600, Alco System, Chiba, Japan) and switching system (model ANI6-A-S, Alco System) which allows the spectrometer to sample the gas from each chamber. Respiratory exchange rate (RER), fat oxidation (FO), carbohydrate oxidation (CO) and exergy expenditure (EE) were calculated from the measured respiratory gas.

### Statistical analysis

All data were analyzed using IBM SPSS statistics 25 software. Significant differences in the values of average were determined using a one-way ANOVA, followed by Tukey’s HSD. Significant differences in the values over time were determined using a two-way repeated ANOVA. Values of *p* < 0.05 were considered statistically significant and all results are presented as mean ± standard deviation (S.D.).

## RESULTS

### Body weight, food intake (FI) and feed efficiency ratio (FER)

At the beginning of experiment, there was no difference in BW. After 8 weeks, however, there was significant difference in BW. The post-BW was significantly lower in EXE+CAP than in SED, but there was no difference in BW between EXE and SED. Interestingly, the total amount of FI was significantly higher in EXE+CAP than the others and there was also significant difference in FI between EXE and SED. Consequently, FER of EXE+CAP was significantly lower than that of SED but there was no difference in FER between SED and EXE (Table 2.).

### The rate of adipose tissue

There was no significant difference in the rate of epididymal and perirenal fats. However, there was a clear trend that EXE+CAP showed the lowest rate of epididymal and perirenal fats. The rate of mesenteric fat was significantly less in EXE+CAP than SED but there was no difference in the rate of mesenteric fat between SED and EXE. Consequently, the rate of total abdominal fat was significantly less in EXE+CAP than SED but there was no difference between SED and EXE (Table 2.).

### Metabolism during exercise

**Fig. 2.**
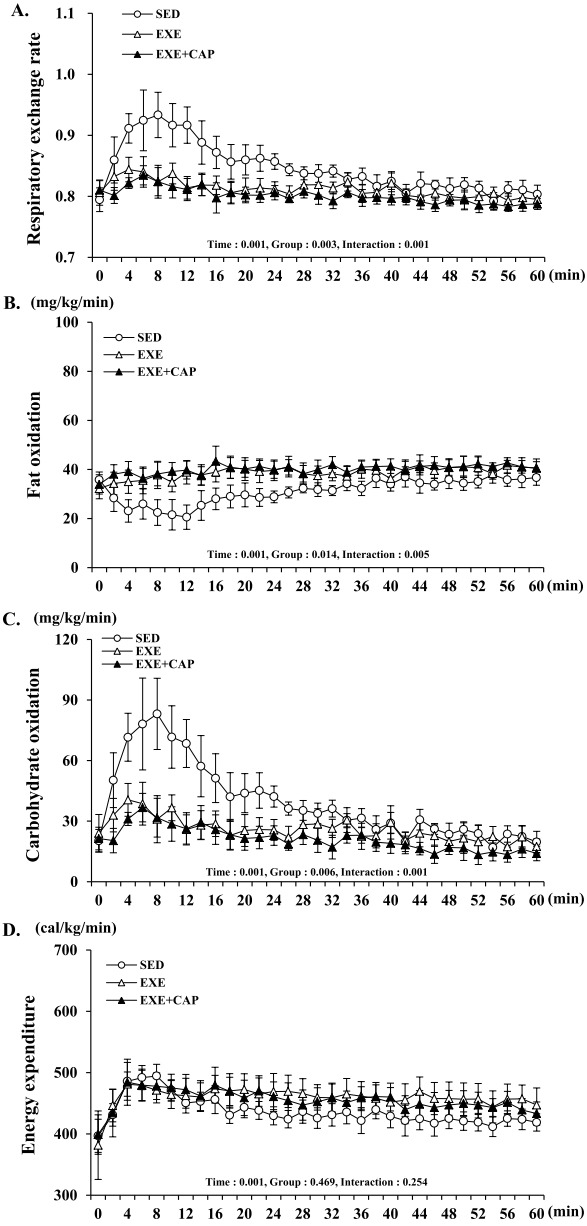
(A), (B), (C) and (D) The changes over time of RER, FO, CO and EE during exercise for an hour, respectively. Values represent the mean ± S.D. (*n* = 8).

**Fig. 3.**
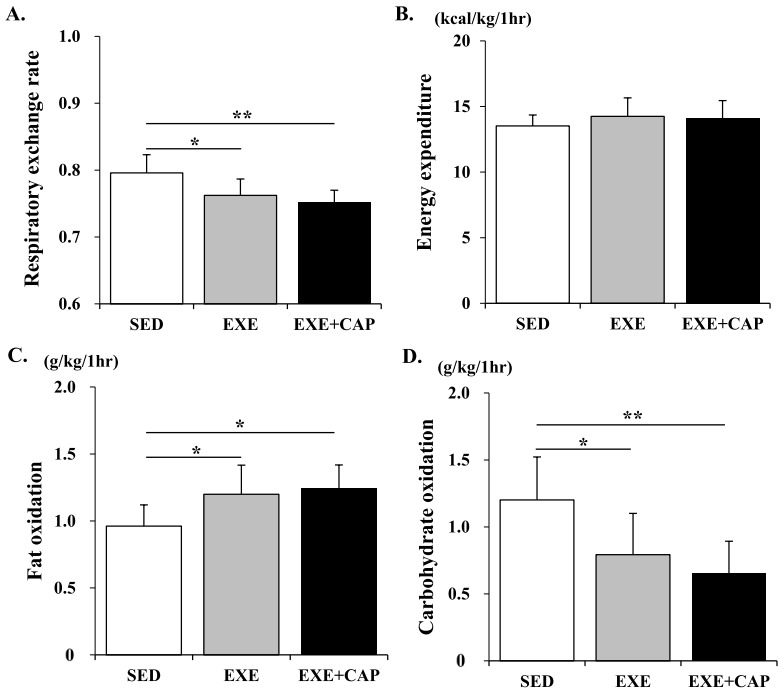
(A) The average of RER and (B), (C) and (D) the total of EE, FO and CO during exercise for an hour, respectively. Values represent the mean ± S.D. (*n* = 8). ^*^p < 0.05; ^**^p < 0.01.

An important feature of exercise training which contributes to the control of body weight is that substrate utilization during exercise could be changed. After 8 weeks, a significant time, group and interaction effects were observed in RER, FO and CO over time (Figure 2A-C.), and there was only a significant time effect in EE over time (Figure 2D.). The average of RER for an hour was lower in EXE than in SED (*p* = 0.025) (Figure 3A.). However, it was significantly much lower in EXE+CAP than SED (*p* = 0.003) (Figure 3A.). Likewise, the total of FO for an hour was much higher in EXE+CAP (*p* = 0.016) compared to the difference between EXE and SED (*p* = 0.045) (Figure 3C.). Subsequently, the total of CO for an hour was significantly much lower in EXE+CAP (*p* = 0.003) compared to the difference between EXE and SED (*p* = 0.028) (Figure 3D.). Consequently, there was no difference in the total of EE for an hour among groups (Figure 3B.). These results indicate that FO capacity which was enhanced by exercise training could be further enhanced by capsiate intake.

## DISCUSSION

The purpose of this study was to investigate whether weight-loss effect of exercise training can be further enhanced by capsiate intake. The results of this study showed that BW was additionally reduced in EXE+CAP compared with EXE despite the higher FI. Furthermore, while there was no difference in the rate of total abdominal fat between SED and EXE, the rate of total abdominal fat was less in EXE+CAP than SED. Thus, we demonstrated that exercise training with capsiate intake was able to additionally reduce BW and the rate of total abdominal fat. Considering the results of metabolism during exercise, this synergetic effect in EXE+CAP may result from FO capacity which was further enhanced by capsiate intake.

Capsiate intake activates sympathetic nervous system [32,33]. Subsequently, catecholamines are released from adrenal medulla which is stimulated by sympathetic nerve. Catecholamines promote fat utilization and thermogenesis [34,35]. In previous studies, epinephrine and norepinephrine levels in plasma were increased by acute treatment of capsiate in humans [12] and rodents [32]. In addition, the mRNA levels of CPT1 and FAT/CD36 (principal transporters of fatty acids) in liver and adipose tissue were increased more than 3-fold by 12 weeks of capsiate intake [36]. Likewise, total FO at rest was increased by 2 weeks of capsiate intake in rodents [15,37] and the average of RER for 30 min at rest was reduced by acute treatment of capsiate in humans [12]. Considering the previous reports, capsiate intake before exercise could play a role as a ‘warm up’ for the oxidation of fat during exercise.

As previously mentioned, FO rate during exercise could be higher depending on metabolic ability [23] or type of exercise [38] despite equal energy expenditure, which can result in a greater loss of body fat. In the previous studies, similarly, Sigal et al. [39] compared the effect of aerobic (FO predominant type) and resistance (CO predominant type) exercises for 6 months on various health indices. There were significant differences in body weight, waist circumference and fat mass between the control and the aerobic training groups but not between the control and the resistance training groups. Also from another study of aerobic and resistance exercise interventions for 12 weeks, body weight and subcutaneous adipose tissue in abdomen were more reduced by 40 % and 70 % respectively in the aerobic group than in the resistance group [40]. Considering the previous studies, likewise, it seems reasonable that greater reduction in the rate of total abdominal fat in EXE+CAP compared with EXE resulted from the additionally enhanced FO during exercise.

Although we demonstrate that the anti-obesity effects of exercise training can be further enhanced by capsiate intake, there may be two limitations in the current study. First, a non-trained group with capsiate intake was not included. The reason why we excluded this group is that our main interest was to explore whether the rate of FO which was increased by exercise training could be further enhanced by capsiate intake or not. Second, we did not analyze the genomics or proteomics related to energy metabolism. Thus, further studies are needed to be done on these perspectives.

In conclusion, the anti-obesity effects of exercise training can be further enhanced by capsiate intake according to the increase of FO during exercise. Therefore, we suggest that capsiate could be a candidate supplement which can additively ameliorate obesity when combined with exercise training.

## Figures and Tables

**Table 1.** The protocol of mild-intensity exercise training.

Weekend	1^st^	2^nd^	3^rd^	4^th^	5^th^	6^th^	7^th^	8^th^
Duration (min)	20	30		40		50		60
Velocity (m/min)	13		14		15		16	
Slope (°)				8				

**Table 2.** The results of body weight, body weight gain, food intake, feed efficiency ratio and the rate of adipose tissue.

		SED	EXE	EXE+CAP
BW (g)	Pre	34.91 ± 1.87	35.28 ± 1.84	34.69 ± 1.48
	Post	47.79 ± 1.54^a^	46.95 ± 4.70^ab^	44.03 ± 1.22^b^
BWG (g/8 wk)		12.88 ± 2.36	11.68 ± 4.51	9.34 ± 1.15
FI (g/8 wk/mouse)		177.7 ± 3.00^a^	188.5 ± 11.1^b^	201.5 ± 3.96c
FER (BWG/FI*100)		7.26 ± 1.42^a^	6.21 ± 2.36^ab^	4.64 ± 0.61^b^
Adipose tissue (mg/bw g)				
Epididymal		37.33 ± 4.74	33.50 ± 9.11	28.53 ± 8.70
Perirenal		15.34 ± 3.99	16.71 ± 6.53	11.11 ± 2.88
Mesenteric		27.40 ± 3.21^a^	25.09 ± 2.92^ab^	22.58 ± 3.65^b^
Total		80.07 ± 7.15^a^	75.30 ± 18.1^ab^	62.21 ± 14.6^b^

Note: Values represent the mean ± S.D. (*n* = 8). Different subscripts indicate a significant difference from each other. p < 0.05
